# Modeling and Implementation of TEG-Based Energy Harvesting System for Steam Sterilization Surveillance Sensor Node

**DOI:** 10.3390/s20216338

**Published:** 2020-11-06

**Authors:** Mateusz Daniol, Lukas Boehler, Ryszard Sroka, Anton Keller

**Affiliations:** 1 Faculty of Electrical Engineering, Automatics, Computer Science and Biomedical Engineering, AGH University of Science and Technology, 30-059 Kraków, Poland; lukas.boehler1@gmail.com (L.B.); ryszard.sroka@agh.edu.pl (R.S.); 2 B. Braun Aesculap AG, D-78532 Tuttlingen, Germany; anton.keller@aesculap.de

**Keywords:** energy harvesting, steam sterilization, IoT, sensor system

## Abstract

The aim of this work is a proof of concept, that medical Internet of Things (IoT) sterilization surveillance sensors can be powered by using the heat during a steam sterilization procedure. Hereby, the focus was on the use of thermo-electrical generators (TEG) to generate enough power for an ultra-low-power sensor application. Power generation requirement of the sensor was 1.6 mW over the single sterilization cycle. The thermal gradient across the TEG has been achieved using a highly efficient aerogel-foam-based thermal insulation, shielding a heat storage unit (HSU), connected to one side of the TEG. The evaluation of the developed system was carried out with thermal and electrical simulations based on the parameters extracted from the TEG manufacturer’s datasheet. The developed model has been validated with a real prototype using the thermal step response method. It was important for the authors to focus on rapid-prototyping and using off-the-shelf devices and materials. Based on comparison with the physical prototype, the SPICE model was adjusted. With a thermal gradient of 12 °C, the simulated model generated over 2 mW of power. The results show that a significant power generation with this system is possible and usable for sensor applications in medial IoT.

## 1. Introduction

### 1.1. Internet of Medical Things (IoMT)

Digital healthcare is a fast-growing area in the medical industry with a substantial potential of delivering secure and high-quality patient care. Many reports suggest that digitalization is becoming a new business opportunity for the healthcare industry involving the Internet of Medical Things, Big Data and automatization. This would lead to cost- and time- efficient workflow of points of care and hospitals by establishing data-driven connection between patients, healthcare professionals, manufacturers, and providers [1,2,3,4]. There are strong indications that bringing the idea of traceable and connected devices to the operating room can improve significantly the efficiency and safety of surgical procedures [5,6]. Moreover, some papers highlight the direction towards digitalization in hospital management systems to create so-called “Smart Connected Hospitals” with sensor nodes and tracking systems. To make this possible, many different technologies are suggested for medical IoT, like Radio Frequency Identification (RFID), ZigBee, Narrow Band Bluetooth and Bluetooth-Low-Energy [3].

### 1.2. Hospital Steam Sterilization and Its Effects on Sensor Systems, Their Power Supply, and Their Specifications

**Figure 1:**
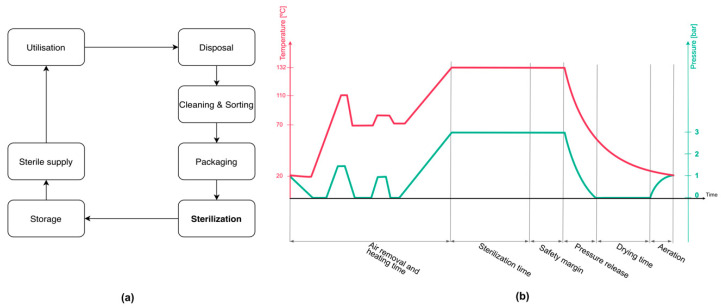
(**a**) Instrument life-cycle process diagram, (**b**) steam sterilization overview.

One of the key requirements for medical electronic devices is to ensure their reliability in daily use in the environment of a healthcare facility. Proper handling and decontamination of these sets is an essential requirement for patient safety, as they can provide a potential route for the transmission of pathogens and bacteria into a patient’s body [7,8,9]. The whole cycle is presented in Figure 1a. After utilization in the operating room, surgical tools are transported to the central sterilization facility where they are sorted, cleaned, and packed for sterilization. Sterilization plays a key role in decontamination of surgical devices. It destroys all microorganisms on the surface of an article or in a fluid to prevent disease transmission associated with the use of that item [10]. There are several different methods of sterilization described and evaluated in the literature [11,12,13,14], the most common method, steam sterilization, is shown in Figure 1b.

The basic principle of steam sterilization is to expose each surgical device to direct steam for a specified time. Thus, four different factors play a key role here: pressure, heat, steam, and time. As a gold standard two different sterilization modes are defined: 121 °C for 15 min and in 132–135 °C for 3–5 min [11,14]. To make this possible, the whole process involves three phases: air removal, injection of saturated steam (sterilization), and the drying phase. During the air removal, pressure oscillations from 0 to 3 bar (absolute values) are generated inside the autoclave. After the residual air is removed, the sterilization phase can begin. The temperature of 121 °C or 132–135 °C, depending on the chosen sterilization mode, is achieved by the injection of saturated steam inside the sterilization chamber under the high pressure of 3 bar. The requirements for the steam sterilization process and autoclave devices are specified in European Standard EN-285 [15]. Autoclave steam sterilization is suited only for objects that can tolerate humidity, high pressure, and high temperature. This makes it currently not acceptable for most embedded electronics. Semiconductor devices can withstand temperatures up to 150 °C in some cases, however, the memory devices that use floating-gate technology (i.e., electrically erasable programmable read-only memory—EEPROM) can be sensitive to such temperature. The energy storage devices, especially rechargeable batteries, cannot withstand such conditions, and need additional protection [16]. Heat resistant power sources are still a big challenge, especially in the case of applications with limited space. One of the possible solutions is to use thermo-electric generators (TEG). Using the Seebeck effect, TEG converts a thermal gradient across its sides into electrical energy. Thermal energy harvesting is a well-known topic, and TEGs have a lot of applications in wide areas of the industry, from aerospace to oil drilling [17,18], however its use in medicine is still very limited and focused mainly on wearable or implantable devices [19,20,21,22,23,24]. The high and variable temperature of steam sterilization suggests considering thermal energy harvesting for powering electronic sensors. Due to the harsh environment of the steam sterilization process and the overall logistics in the hospital there are some limitations and requirements to the project of such a sensor node:

- Maintenance free: absolute requirement due to the work overload of hospital staff.
- Harsh environment resistant and safe: the whole design should assure increased safety and reliability against steam sterilization environment.
- The size of the device needs to fit the design of the surgical container.

Based on these requirements, the authors developed a concept of energy harvesting system for an autonomous sensor node for steam sterilization surveillance. The system consists of power generation and data acquisition and processing modules. Within the scope of this article the modeling and prototype validation of power generation module is described.

## 2. Materials and Methods

### 2.1. Power Requirements of the Sensor System

**Figure 2:**
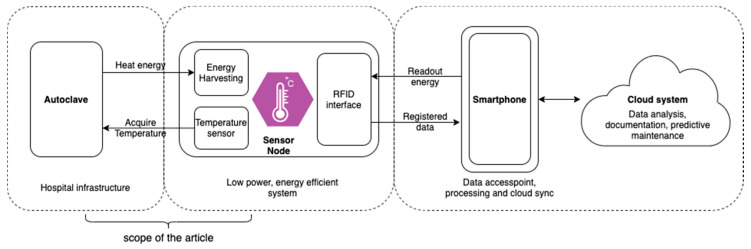
System architecture concept.

To set the initial requirements for the power generation module, the design of the sensor node had to be established using low power technologies where it was possible. To provide data persistence and low power acquisition, the passive readout RFID-enabled microcontroller with Ferroelectric Random Access Memory (FRAM) was used as a data processing unit. To provide an easy to use and low-cost interface, a smartphone can be used as an interface between the sensor and the cloud system. The whole concept of the system is presented in Figure 2.

A sensor node is powered using the heat from the steam sterilization performed in the autoclave. The temperature sensor registers the temperature in the autoclave. Using radio field energy, harvesting the data can be then sent to the smartphone device RFID technology (13.56 MHz, ISO15693 compliant). Smartphone serves as a connection point to the cloud systems. Due to its matching specifications, the RF430FRL152H chip has been selected. For the development phase, an external thermistor was used. For a first evaluation, 10 test cycles lasting 10 min each were carried out using custom-made software. Electric parameters such as power, energy, and current consumption were registered and served as an initial requirement for the further energy harvesting module design. For registration purposes Texas Instruments EnergyTrace technology and MSP-FET programmer were used with a sampling rate of 1 kHz. 

### 2.2. Modeling of the TEG-Based Self-Powered System

The main technical challenge of this study is to provide a thermal gradient across the TEG module sufficient for powering a sensor node, while keeping the size of the energy-harvesting module as small as possible. Previous research showed that a thermal insulated metallic heat storage unit (HSU) connected to the TEG results in a significant heat difference during a steam sterilization cycle [25]. A thermal insulation is placed between the prototype casing and the HSU. This assures that the only thermal bridge between the HSU and autoclave environment is over the TEG module.

#### 2.2.1. Thermal Insulation Materials Selection

Proper insulation plays a key role in forming a thermal gradient across the TEG. The current state of the art focuses on the use of epoxy resin or high temperature silicone encapsulations. The most critical component of electronic systems is the energy storage device [26,27,28,29]. Therefore, many studies focus on the thermal protection of high temperature batteries which are able to withstand up to 85 °C. In the work of George et al. [30], two epoxy resins and one heat resistant silicone was used to test the effect of steam sterilizations on batteries. Material parameters of both epoxy resins and silicone are listed in the Table 1.

In three cases, 225 sterilization cycles were processed without failure. Hereby, 3 mm OD2002 epoxy resin as well as 1 and 5 mm Mold Max 60 silicone were used. This test shows the possible use of epoxy resin and silicone to thermally protect electronic devices for a multitude of autoclave cycles. However, in most cases, failures in the encapsulation integrity or thermal damage of the energy storage system led to a negative result. Therefore, the reliability of this insulation cannot be guaranteed. This conclusion was also made by a previous work [31]. Hereby, the thermal insulation of a special developed epoxy resin was evaluated. Despite the good heat insulation, the non-elastic structure can lead to cracks due to the fast temperature and pressure changes during the steam sterilizations. In addition, small air encapsulations cause a strong internal stress to the insulation, leading to damages.

For this reason, a new approach for insulating electronics was made. A material known for its very low thermal conductivity but almost unknown in the medical industry is aerogel [32]. This material group can be divided into the most common inorganic, organic and inorganic–organic aerogels. The combination of alkoxides with metal oxides like silicone creates a nanoporous structure with pores between 5 and 100 nm. Hereby, up to 99.8% of the material is air, which leads to a very low density and high specific surface area. These properties enable multiple possible applications in science like Cherenkov counters, aerospace insulations, or thermal super-insulations.

Given its physical properties, aerogel brings many benefits like a very low thermal conductivity (20 $\frac{\mathrm{mW}}{m\;\cdot \;K}$), a density of 10.8 kg/m³ and a high mechanical strength, making it a promising insulation material for medical sterilizable applications. Moreover, ease of application of aerogel foam and its high maximal operating temperature of 150 °C enables insulating even complicated and irregular structures, still providing similar thermal properties. 

In the previous research it was found out that a highly efficient and low-profile encapsulation is possible, however, due to the pressure changes during steam sterilization, an outer encapsulation using epoxy resin needs to be used, protecting the fragile aerogel [31]. These results were carried out in this work.

#### 2.2.2. Prototype Design

**Figure 3:**
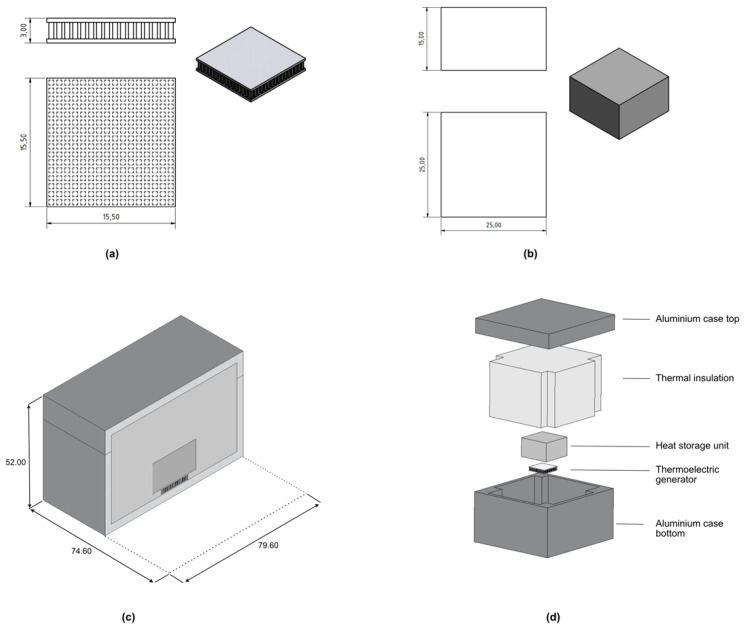
(**a**) A thermoelectric generator with its dimensions used for this study, (**b**) a steel heat storage unit (HSU), (**c**) cross-section of the prototype and 3D module with the outer sizes, (**d**) exploded view of used 3D model as well as a prototyped module. All the sizes are in mm.

As a first step, a thermal simulation had to be performed, calculating the resulting heat difference and therefore the resulting power using 23.5 mm of aerogel-based insulation from the side walls of the module. For comparing the simulation results to real tests in the next step, the used 3D model was designed like the planned prototype. Hereby, a TEG module is connected to the steel HSU using thermally conductive paste. Both used TEG and HSU are shown in Figure 3a,b.

Due to the fragile structure of the selected aerogel-based insulation, an aluminum case was selected to enable heat transition to the internal modules. To increase the thermal flux from the applied heat, the TEG is connected to the metal case and to the HSU with a thin layer of conductive grease in between. For generating a heat difference, the HSU and upper part of TEG are embedded into the insulation. A cross section of the created 3D model with its sizes and an exploded view are shown in the Figure 3c,d.

#### 2.2.3. Simulation Setup

The aim of the simulations was to estimate the heat gradient response for the thermal step on the hot and cold side of the TEG inside the prototype. This, after prototype validation, would allow simulation of the thermal gradient through the TEG during steam sterilization. The simulation is carried out using OpenFoam solvers—an industrial open source toolbox for heat transfer simulation. For the given simulations the MUMPS solver was selected with SCOTCH renumbering method and a Theta parameter equalling 0.57. The prepared model is exposed to a thermal step signal to estimate the thermal response and the reached temperatures inside the model, especially on the both sides of the TEG. The material parameters are specified in the Table 2. Simulation has been running for 130 min (7800 s) with time step length of 120 s. The initial temperature of the model for the simulations was set to 100 °C, the same as previously measured in the real prototype. Because the aim of the simulation was to know the temperature distribution inside the model, the heat exchange between the outer case and the air was not the subject of this simulation. The temperature of the aluminum case was known from the prototype measurements and was set as a boundary condition. All the material parameters have been taken from standard material specification sheets.

Due to the needed temperature difference of the two sides of the TEG for generating power, the simulation is stopped when the temperature difference on both sides of the TEG is less than 3 °C. 

#### 2.2.4. Modeling of the TEG

**Figure 4:**
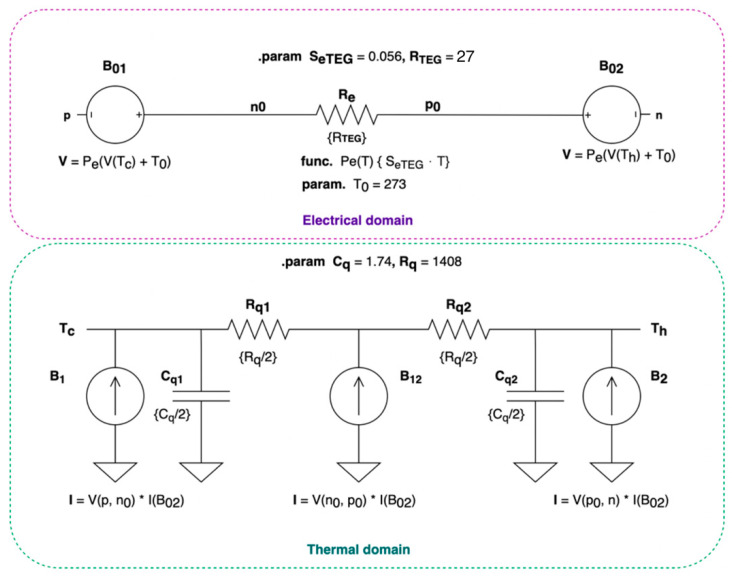
Kubov’s thermo-electrical generators (TEG) electric circuit representation with SPICE directives. The upper part is a model of electrical domain of the TEG while the bottom part models the thermal behavior of the TEG.

In the presented application the heat flux is bi-directional between the HSU and the variable temperature of the prototype walls. During the steam sterilization procedure, the heat flux is directed towards HSU, and after the steam sterilization—while cooling down—it is the opposite way. Some studies had described similar setups, such as powering systems for autonomous sensor node in aircrafts [33]. Several different modeling approaches have been investigated, including very interesting system approaches of Dziurdzia [34] and Mirocha [35]. Some authors present very comprehensive models [36], depending on the environmental conditions, however a big drawback of such models is the need to estimate the parameters, which sometimes are not known from the TEG manufacturer’s datasheet. Due to that, the biggest emphasis of this study was to provide a possibly easy to use and intuitive way for estimation of the output of the TEG-based energy harvesting system, limiting possibly the need for additional measurements and using only the manufacturer’s datasheets. Thus, the Kubov’s model [37] was selected for further investigation, as it is very intuitive and easy to use, using only four parameters as an input and focused on the electrical domain, which is also a main scope of this study. It have been also proven by a study by Afghan and Géza describing simulation and analysis of an energy harvesting system for wearable applications [38]. The model itself is presented in Figure 4. There are two parts of the Kubov’s model—electrical and thermal. In the thermal part, the current is an analog of thermal power, and voltage represents the temperature. In the electrical part, the voltage generated on the p-n terminals is dependent on the temperatures *T_h_* and *T_c_* of the thermal part inputs. Resistor Re represents TEG internal electrical resistance, while resistors *Rq* and capacitors *Cq* represent thermal resistance and capacitance of the module, respectively. Current sources *B1, B2, B12* represent compliant thermal power sources dependent on the voltage drop across the TEG. A detailed description of the Kubov’s model is presented in his paper [37].

All of the needed parameters have been estimated based on [39] and the generalized approach of Kubov’s model. Electrical resistance of the TEG module can be calculated as follows:

(1) $$R_{TEG}=\frac{V_{load}^{2}}{W_{load}}$$

where *V_load_* is a matched voltage and *W_load_* is a power at the matched load.

Or in the case of the thermoelectric cooler:

(2) $$R_{TEC}=\frac{V_{opt}^{2}}{2\;Q_{cold}}$$

where *Q_cold_* is a maximum performance for Δ*T* = 0, and *V_opt_* is a maximum output voltage. Seebeck coefficient, depending on available data from the manufacturer’s datasheet can be expressed as:

(3) $$S_{eTEG}=\frac{2V_{load}}{T_{hot}-T_{cold}}$$

or:

(4) $$S_{eTEC}=\frac{V_{opt}}{T_{hot}+T_{0}}$$

where *T_0_* = 273 K, *T_hot_* is a temperature on the hot side, and the *T_cold_* is a temperature on the cold side from the manufacturer’s datasheet.

Thermal resistance of the TEG can be calculated as follows:

(5) $$R_{q}=\frac{\Delta T_{max}}{I_{max}U_{max}}\;\frac{2T_{hot}}{T_{hot}-\Delta T_{max}}$$

The thermal volumetric capacity *Cq* can be calculated from the TEG datasheet based on the materials from which the TEG is composed. This has been taken from [40]. After applying the material properties to volumetric data taken from the TMG-127-0.4-1.6 datasheet, the following volumetric heat capacity was calculated:

(6) $$C_{q}=1.74\;\left[\frac{J}{K}\right]$$

A study by Nesarajah and Frey [41] proves that in the temperature range 0–100 °C, on which the TEG in the described prototype is mostly exposed during steam sterilization, a thermoelectric cooler (TEC) can be used as the thermoelectric generator, so the given calculations can be applied both to TEC and TEG models of the manufacturers, making this analysis more universal. 

Having developed a model of the TEG, a simulation under the given thermal gradients across the TEG could be performed. The step function has been applied to the SPICE model of the TEG having a temperature on the hot and cold sides of the TEG as an input of the simulation. The model analysis has been performed in the range of Δ*T* from 5 °C to 22 °C with the 1 °C step. *T_cold_* and *T_hot_* input parameters have been set to the values measured from the prototype, where the given Δ*T* occurred, while performing a thermal step response test. The open-circuit voltage (*V_occ_*) was measured across the n-p terminals of the model. The results were then compared with data acquired from the prototype.

### 2.3. Practical Implementation of the TEG-Based Energy Harvesting System

**Figure 5:**
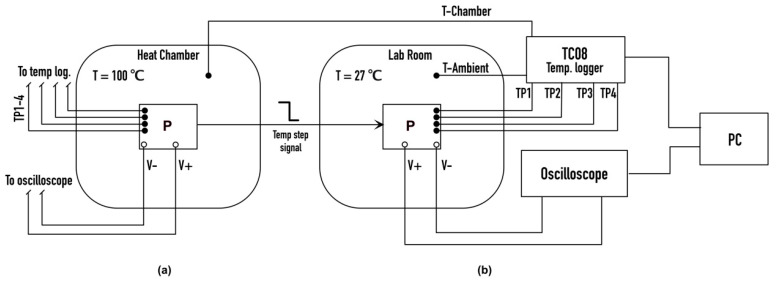
Scheme of the conducted experiment with the prototype (P). Measurement setup used in the heat chamber (**a**) and while simulating temperature step signal in the laboratory room (**b**).

**Figure 6:**
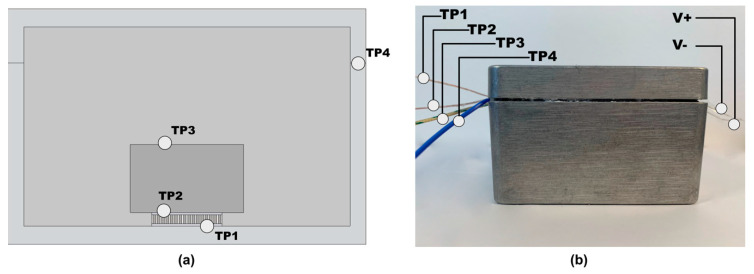
Test points of the model (**a**) and prototype (**b**) to compare the heat distribution in a simulated environment in comparison to the prototype tests in the heat chamber.

A prototype of the described energy harvesting system was built. The aim of this prototype was to validate the thermal simulations and the TEG electrical model. Four different test points were defined to compare the simulation results with the experimental measurements. The experiment scheme is shown in the Figure 5. The prototype (P) was heated up to 100 °C inside the thermal chamber, after this it was placed out of the chamber in the laboratory room with ambient temperature of 27 °C, simulating the step temperature signal. All the test points are shown in Figure 6a,b. Test point 1 (TP1) was placed on the connection between the “hot” side of the TEG and the aluminum case of the prototype, test point 2 (TP2) was placed between the “cold” side TEG and the HSU, test point 3 was placed on the HSU, and test point 4 was placed on the aluminum case of the model.

The thermocouples were placed in TP1-3 to measure the heat distribution among these surfaces in the prototype. The thermocouple TP4 was placed within the wall of the prototype. The measurements in all test points were performed using a PICO PicoLog TC-08 (Pico Technology, St. Neots, UK) registration device with attached 4 K-Type IEC584-2 (1982) standard compliant thermocouples with ±1.5 °C accuracy. The measurement uncertainty of the TC-08 device can be defined as follows:

(7) $$E_{TC08}=0.2\;\frac{T_{max}-T_{amb}}{100}+0.5\;{}^{\circ}C$$

where the *T_max_* is maximal measured temperature, and the *T_amb_* is an ambient temperature of the TC08 temperature logger.

Therefore, the overall measurement error can be defined as follows:

(8) $$E_{m}=\sqrt{E_{TC08}^{2}+E_{K}^{2}}=\sqrt{{\left(0.2*\frac{105-25}{100}+0.5\right)}^{2}+1.5^{2}}=\pm 1.28\;{}^{\circ}C$$

where $E_{K}$ is an accuracy of the K-Type thermocouple.

The sampling frequency of the TC-08 temperature logger was set to 1 Hz. The tests lasted 130 min. After data registration the temperature curves were compared with the simulation results. The voltage generated from the TEG was registered on output terminals shown in Figure 6b (V−, V+). Registration was made using PicoScope 2406B digital oscilloscope (Pico Technology, St. Neots, UK), with bandwidth of 50 MHz, 8 bits resolution, and 32 MS memory.

## 3. Results

Registered power consumption of the given sensor application has been very low—0.79 mW on average. However, due to the reliability and safety requirements we assume that the power system needs to deliver double the power needed at its peak consumption. Therefore, the power delivery requirement for the thermoelectric generator (TEG) was set to 1.6 mW.

**Figure 7:**
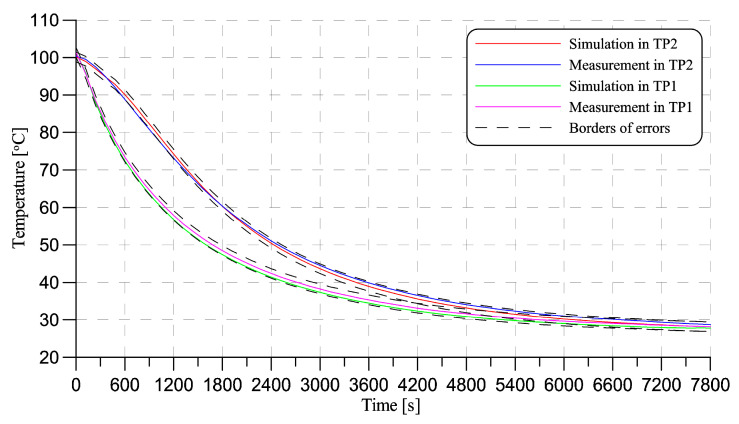
Comparison between simulated results and thermal measurements using K-Type thermocouple in test-points TP1 and TP2.

Figure 6 depicts the placing of the test points (6a) and the overview of the prototype with attached thermocouples (6b). The comparison of the simulation and measurement results are shown in Figure 7. It can be seen that the simulated results differ from the measured ones, however the difference is still in the boundaries of possible acquisition inaccuracies. This indicates that a simulated environment can be used for further analysis using the thermal signal from the steam sterilization process.

**Figure 8:**
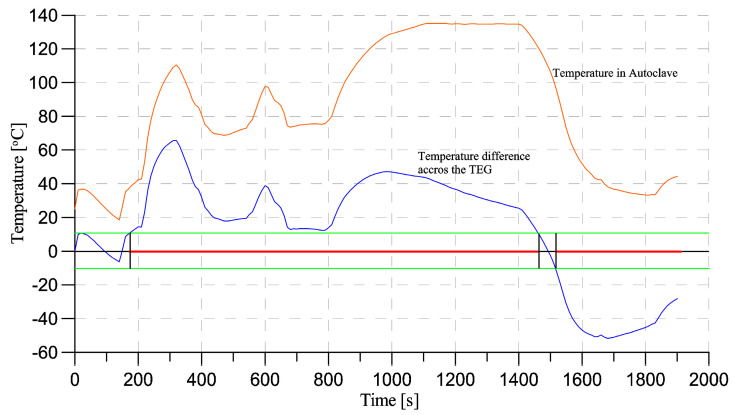
Temperature inside the autoclave and on the surface of the walls of the prototype (orange line) during single steam sterilization procedure, and corresponding thermal gradient across the TEG module inside the prototype (blue line).

Based on the acquired results, it was possible to assume that the model is accurate enough to conduct further analysis of the steam sterilization procedure in the case of its thermal properties. The simulation settings remained the same. The boundary conditions were set based on previously made calorimeter measurements to fixed temperature values on the prototype walls. The specification of the saturated steam sterilization process is to ensure that the surgical instruments surface temperature is compliant with the standards of the procedure. The measurements taken inside the autoclave are presented in Figure 8 (orange line); this measured temperature in test point TP4 was set as a boundary condition for simulation. The second curve shown in Figure 8 (blue line) represents the simulated thermal gradient across the TEG during the steam sterilization procedure (Δ*T* = TP1-TP2). The green lines represent the minimal thermal gradient needed for sufficient power generation for the sensor node. The red line indicates the time where enough power is generated over the whole sterilization procedure. As it is shown, the peak gradient value exceeds 60 °C. For most of the time (89% of the overall time of the sterilization), the thermal gradient of 12 °C is reached. What is more important is that after the sterilization cycle, the power is still generated, as the heat transfer is in the opposite direction—from the HSU to the environment, which is shown from 1500 s (t > 1500 s). 

**Figure 9:**
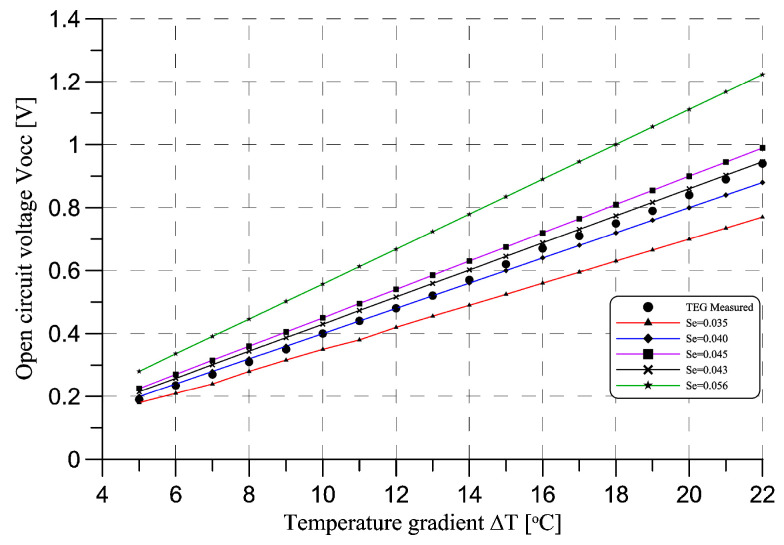
Comparison of different model adjustments and the acquired data from the TEG used in the prototype.

As it can be seen in Figure 9, the initial parameters of the TEG model calculated from the manufacturer’s data (Se = 0.056) do not converge with the measured values. Thus, the series of adjustments were made to find the optimal value of Seebeck’s coefficient parameter. The value of 0.043 was found as the one that fits the measured TEG parameters in the selected thermal gradient from 5 °C to 22 °C. This can be caused by several different reasons, including aging of the module, working conditions inside the prototype, interaction with other components, i.e., aerogel and other chemical substances used to insulate the whole module could interact negatively with the TEG. Moreover, some inaccuracies during the measurements and simulations could affect the results. The most probable explanation is aging of the module combined with micro-cracks inside the Peltier elements of the module. However, such an investigation is out of the range of the scope of the article, thus the value of 0.043 was taken to perform further power generation simulations.

**Figure 10:**
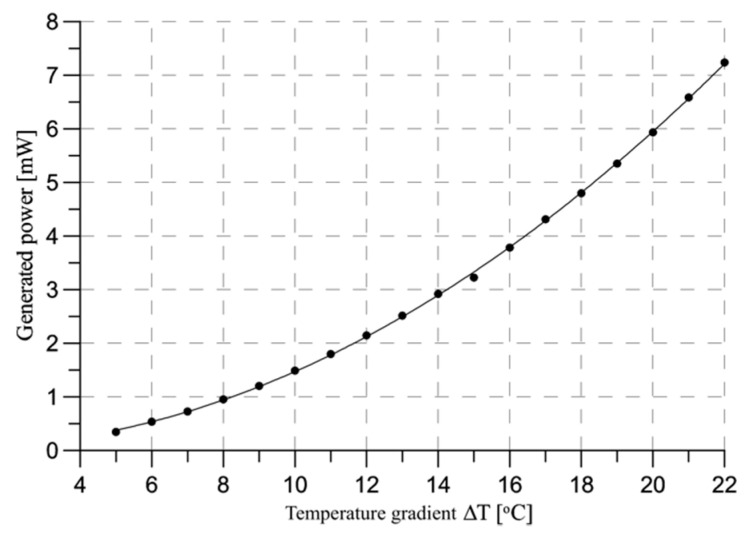
Power generated on the different thermal gradients with matched load.

As it can be seen in Figure 10, the power generated by the TEG module inside the prototype provides 2.14 mW of power with a matched resistance from a thermal gradient of 12 °C which is present in most of the time of steam sterilization cycle, and still exceeds the initial requirements of the sensor node.

## 4. Discussion

From a thermal analysis perspective, it was proven that using aerogel as an insulation material according to a proper design of the module can create the thermal gradient across the TEG. Moreover, the heat-step response simulation results converged with the measured values from the real prototype experiment. This allowed us to simulate the steam sterilization procedure and use this simulation for further development. Thermal gradient with the peak reaching over 60 °C was achieved during the steam sterilization procedure, and for most of the time the gradient of at least 12 °C was reached. The main limitation of our study is that even though we performed the measurement of the temperature of the aluminum walls of the prototype, it was not possible to register the temperatures inside the prototype during the steam sterilization. However, based on the heat-chamber experiments, it was confirmed that the simulated values of the temperatures inside the prototype converge with the measured ones.

In the case of the electrical part of the design, the TEG module TMG-127-0.4-1.6 from Ferrotec-Nord (Moscow, Russian) proved that it can be used for powering the sensor node according to the initial requirements. As it was presented in Figure 8, the thermal gradient across the TEG is over 12 °C for most of the time, ensuring that the module will produce at least 2 mW of power. With sensor module consumption it is still possible to store the additional power in a backup energy storage system. It is worth mentioning, however, that this TEG has relatively high internal resistance of 27 Ω, which limits the possible power generation and causes this TEG to be suitable only for super-low power applications. Additionally, power generated by the TEG module cannot be directly used by the sensor electronics. The use of boost converter and voltage stabilizer is a need to provide a reliable power source for the chip. This work is a further development of the idea presented by the authors, however different technologies and a passive data storage system concept is investigated in this study in comparison to [25]. Another issue is to handle both the positive current generated by the energy harvesting module during steam sterilization procedure as well as a negative current generated after the whole procedure, when the module is cooling down and the heat flux is in the opposite direction. This might require using an interface circuit i.e., as described by Toh [42]. Due to the lack of similar works in the literature, it is hard to make any comparison of obtained results, as the topic of energy harvesting in medicine is not vastly exploited, and the papers mostly focus on wearable devices or in-body implantable designs. In comparison with the author’s previous work, the size of the prototype is significantly smaller, however, this could be due to the lower power requirements of the sensor node. In the previous work, authors focused on powering the Bluetooth Low Energy device, while in this paper only the RF430 passive RFID chip had to be powered. This resulted in using a single TEG module in the current work in comparison to two TEG modules connected in series in the previous prototype [25].

## 5. Conclusions

Within this work a model analysis of the energy harvesting from steam sterilization was performed. The model was developed within the thermal and electrical domain, and a 3D model of a prototype was made for the simulations. Developed models were validated with the real-world prototype using the thermal step response. This confirmed that it could be used to model a real steam sterilization procedure. The model and a prototype showed that the initial power requirements of the sensor node were met. Results of this paper can be used for further optimizations of the prototype, especially to work on the miniaturization. Another topic that needs to be covered in the upcoming research is to develop an interface circuit and power management system for the possible wide range of the sensor types. 

## Figures and Tables

**Table 1:** Physical parameters of the currently used heat and pressure insulation materials for steam sterilizable electronics.

Material Parameters	Henkel FP4460 Epoxy	Epotek OD2002 Epoxy	Mold Max 60 Silicone
Modulus of Elasticity [G Pa]	10.8	1.815	0.006
Max. Operating Temperature [°C]	150	225	294
Thermal Expansion [ppm/°C]	20	46	260
Thermal Conductivity [W/(m·K)]	0.68	0.3	0.21
Thickness [mm]	1, 3, 5	1, 3, 5	1, 3, 5

**Table 2:** Material parameters used for the heat transfer simulations of the prototype 3D model.

Material	Density [kg/m^3^]	Thermal Conductivity [W/(m·K)]	Specific Heat [J/(kg·K)]
Aluminum	2700	235	897
Aerogel	120	0.025	1500
Steel	7870	60	480
Bi_2_Te_3_	7740	1.6	200
Al_2_O_3_	3720	25	880
